# Effects of Flow Distributor Position and Loosener Configuration on Particle Flow Behavior in a Hydrogen-Based Direct Reduction Shaft Furnace

**DOI:** 10.3390/ma19102160

**Published:** 2026-05-21

**Authors:** Qingbin Xue, Haotian Liao, Qiqiang Zhao, Aibing Ji, Dongsheng Huang, Guolei Zhao, Chunhe Jiang, Jianliang Zhang, Kejiang Li

**Affiliations:** 1 School of Metallurgical and Ecological Engineering, University of Science and Technology Beijing, Beijing 100083, China; 2 MCC Huatian Engineering & Technology Co., Ltd., No. 18, Fuchunjiang East Street, Nanjing 210019, China; 3 Technical Support Center for Prevention and Control of Disastrous Accidents in Metal Smelting, University of Science and Technology Beijing, Beijing 100083, China; 4 School of Chemical Engineering, The University of Queensland, St. Lucia, QLD 4072, Australia

**Keywords:** hydrogen-based shaft furnace, discrete element method, internal structure, particle flow behavior

## Abstract

This study investigates the effects of flow distributor placement and loosener configuration on particle-flow behavior in a hydrogen-based direct reduction shaft furnace using the discrete element method (DEM). A three-dimensional industrial-scale furnace model based on a MIDREX-type geometry was established, and four representative structural configurations were examined by varying the flow distributor position and loosener setting. The results show that flow distributor placement is the dominant factor controlling particle descending behavior and particle-flow uniformity. When the flow distributor was located in the cooling zone, the flow uniformity index reached 0.875, which was 40.9% and 20.9% higher than those for the transition–cooling interface and transition-zone configurations, respectively. Particle trajectory analysis indicates that the effect of flow distributor position is mainly confined to the region above the device, with limited influence on the lower burden trajectory. Although the loosener has little effect on particle-flow uniformity, it significantly suppresses particle degradation. Under the transition-zone flow distributor configuration, the predicted powder formation ratio decreased from 3.89% to 2.97% after introducing the loosener, corresponding to a relative reduction of 23.7%. Overall, among the four representative configurations investigated in this study, positioning the flow distributor in the transition zone while retaining the loosener provides a more balanced compromise between burden-flow regulation and powder suppression for shaft furnace design and industrial operation.

## 1. Introduction

The steel industry is under increasing pressure to reduce carbon emissions and accelerate its low-carbon transition. Compared with the conventional blast furnace–basic oxygen furnace route, hydrogen-based direct reduction has attracted extensive attention because hydrogen can partially or even completely replace carbonaceous reductants, thereby substantially decreasing CO_2_ emissions [1,2,3]. In hydrogen-based shaft furnace processes, iron oxides are reduced mainly by hydrogen to produce direct reduced iron (DRI), offering a promising pathway toward cleaner ironmaking [4,5,6]. As representative projects such as HYBRIT continue to demonstrate the feasibility of hydrogen metallurgy, hydrogen-based shaft furnaces are becoming an important technological option for sustainable steel production [6].

A hydrogen-based direct reduction shaft furnace is essentially a gas–solid moving bed reactor in which burden descent, gas flow, heat transfer, and reduction reactions occur simultaneously [7,8]. Therefore, stable and efficient furnace performance depends not only on reaction kinetics, but also on the internal flow behavior of burden materials. Owing to the typical top-wide and bottom-narrow geometry of the shaft furnace, particles often exhibit non-uniform descending behavior, with the central region descending faster than the peripheral region. This may lead to funnel flow, uneven residence time, heterogeneous burden distribution, and even unstable furnace operation [9,10,11,12]. In addition, non-uniform particle flow may aggravate local stress concentration and mechanical degradation of pellets, thereby increasing the risk of fines generation during operation. To regulate burden flow, structural components such as flow distributors and looseners are commonly introduced in shaft furnaces. However, their combined effects on particle flow behavior under hydrogen-based direct reduction conditions have not yet been fully clarified.

Previous studies on direct reduction shaft furnaces have mainly focused on hydrogen reduction kinetics and numerical modeling. In terms of reaction mechanisms, substantial progress has been made in understanding the effects of particle size, gas composition, and temperature on the hydrogen reduction behavior of iron oxides [13,14,15,16,17,18]. In terms of reactor-scale simulation, continuum models based on computational fluid dynamics (CFD) have been widely applied to describe gas flow, temperature evolution, and reaction behavior in shaft furnaces [8,19].

To address this limitation, the discrete element method (DEM), originally proposed by Cundall and Strack [18], has become an effective tool for investigating granular flow in metallurgical reactors. DEM can provide detailed information on particle trajectories, velocity distribution, collision behavior, and contact forces, and has been increasingly applied to direct reduction and related shaft furnace systems [17,18,20,21,22,23,24]. Existing DEM studies have shown that structural and burden-related parameters, such as lump ore ratio and particle shape [21], shaft furnace wall angle [22], and diversion cone structure [23,24], can significantly affect solid flow patterns. However, due to computational limitations, most of these studies have focused on a single structural factor, simplified operating conditions, scaled-down furnace models, or enlarged particles [17,21,22,24]. As a result, the combined influence of flow distributor position and loosener configuration on burden descending behavior in a hydrogen-based direct reduction shaft furnace remains insufficiently understood.

In particular, how these internal configurations jointly regulate particle descending velocity, flow uniformity, trajectory evolution, stress distribution, force-chain characteristics, and fines generation has not yet been systematically clarified. This issue is important because internal structure optimization should not only improve flow organization, but also suppress excessive local stress and particle degradation. Therefore, this study established a three-dimensional DEM model of a hydrogen-based direct reduction shaft furnace under continuous charging and discharging conditions. Different configurations were considered by placing the flow distributor at the transition zone, at the interface between the transition and cooling zones, and in the cooling zone, as well as by comparing cases with and without the loosener. The effects of these internal structural configurations on particle descending velocity, flow uniformity, particle trajectory, stress distribution, force-chain evolution, energy dissipation, and powdering tendency were systematically analyzed. The results are expected to provide theoretical guidance for optimizing the internal structure of hydrogen-based shaft furnaces and for achieving a better balance between flow uniformity, operational stability, and fines suppression.

## 2. Methodology

### 2.1. Discrete Element Method

The discrete element method (DEM) [18] was employed to simulate the granular flow behavior in the hydrogen-based direct reduction shaft furnace. In DEM, each pellet is treated as an independent discrete body, and its translational and rotational motions are calculated by solving Newton’s second law of motion at every time step. Through continuous updating of particle positions, velocities, and contact states, DEM can capture the dynamic evolution of burden flow inside the shaft furnace and provide particle-scale information that is difficult to obtain experimentally. Such information includes particle trajectories, descending velocities, contact forces, force-chain structures, and collision-related energy dissipation.

**Figure 1:**
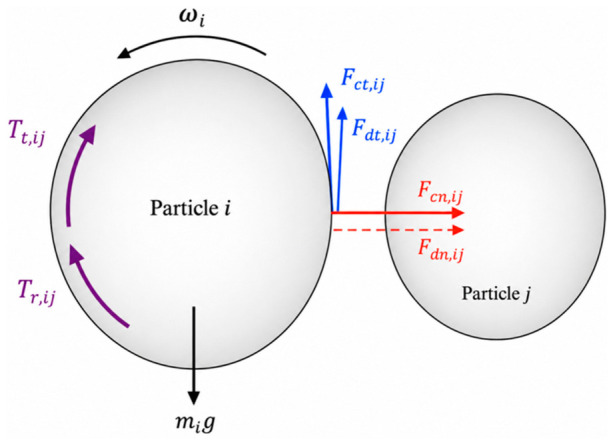
Schematic diagram of the contact forces and torques between two particles in the DEM model.

In this work, the DEM model was established with several simplifying assumptions. The pellets were treated as spherical, dry, non-cohesive particles with uniform size and material properties. The gas phase, heat transfer, and reduction reactions were not considered, because the present study focuses on the mechanical regulation of burden flow by internal structures. The furnace wall, flow distributor, and loosener were treated as rigid boundaries, and particles were charged from the top and discharged from the bottom outlet under gravity. Particle–particle and particle–wall interactions were calculated using a Hertz–Mindlin contact model with rolling friction. To clarify the force system used in the DEM formulation, Figure 1 shows a schematic diagram of the contact forces and torques between two particles. In this model, the translational motion of a particle is governed by the normal contact force, normal damping force, tangential contact force, tangential damping force, and gravitational force, while the rotational motion is governed by the tangential torque and rolling-friction torque. Based on this contact model, the translational and rotational motions of each particle are described by the force and torque balances given in Equations (1) and (2), respectively.

(1) $$m_{i}\frac{dv_{i}}{dt}=\sum_{j=1}^{k_{i}}(F_{cn,ij}+F_{dn,ij}+F_{ct,ij}+F_{dt,ij})+m_{i}g$$

(2) $$I_{i}\frac{d\omega_{i}}{dt}=\sum_{j=1}^{k_{i}}(T_{t,ij}+T_{r,ij})$$

Particle motion is determined by both its intrinsic properties and interaction state. The translational behavior of particle *i* is governed by its mass (***m_i_***), velocity (***v_i_***), and the number of particles in contact with it (***k_i_***). The forces acting on the particle include the normal contact force (***F_cn,ij_***), normal damping force (***F_dn,ij_***), tangential contact force (***F_ct,ij_***), tangential damping force (***F_dt,ij_***), and gravitational force (***m_i_g***). The rotational motion is governed by the moment of inertia (***I_i_***) and angular velocity (***ω_i_***), while the corresponding torques arise from the tangential contact force (***T_t,ij_***) and rolling friction resistance (***T_r,ij_***). The resultant force and torque acting on each particle are obtained by vector summation of all interaction components. Detailed formulations of these force and torque terms can be found in previous studies [25,26,27].

### 2.2. Simulated Setup

A three-dimensional industrial-scale shaft furnace model, with a height of approximately 30 m and a diameter of about 6 m, was established using the open-source DEM software LIGGGHTS (version V3.X.). The reference furnace geometry was constructed according to the structural characteristics of an industrial MIDREX-type shaft furnace and previously reported shaft furnace models [17]. The material properties and contact parameters listed in Table 1 were adopted from our previously published DEM studies on direct reduction shaft furnaces and related granular-flow systems [17,21,22,23], in which these parameters were shown to reasonably reproduce the main particle-flow characteristics. To reduce computational costs, the pellet size was increased compared with actual industrial pellets, following the particle-scaling treatment commonly used in large-scale DEM simulations [23,24]. In all cases, the pellet diameter was set to 96 mm. It should be noted that particle-size enlargement may influence the absolute values of particle descending velocity, contact force, local packing structure, and collision-related degradation because the number of particles and contact network differ from those under real pellet-size conditions. Therefore, the predicted velocity, flow uniformity index, and degradation ratio should be interpreted mainly as comparative indicators rather than direct quantitative industrial-scale values. Since all cases were calculated using the same pellet diameter, material properties, contact parameters, and boundary conditions, the relative differences among different flow distributor positions and loosener configurations remain comparable.

**Figure 2:**
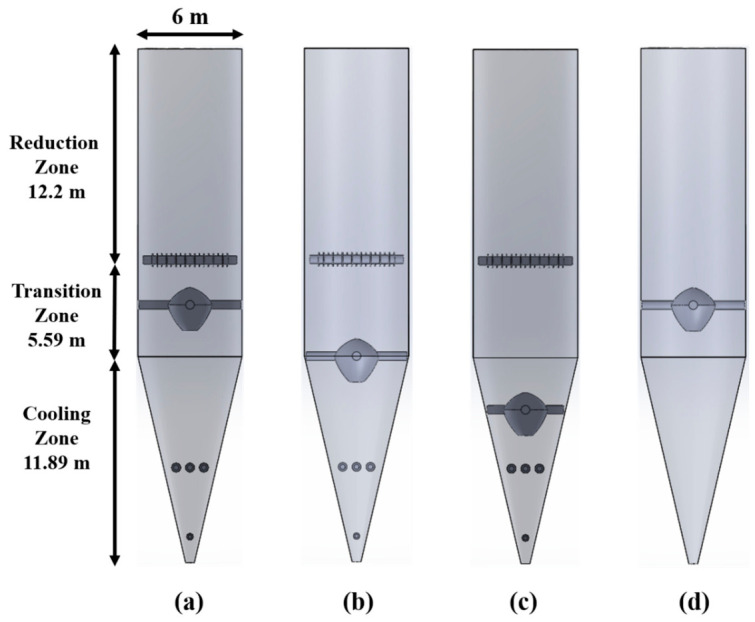
Planar screenshots of the three-dimensional shaft furnace models under different internal structural configurations: (**a**) Case 1, (**b**) Case 2, (**c**) Case 3, (**d**) Case 4.

The internal structure used to guide and redistribute the descending burden is referred to as the “flow distributor” throughout this manuscript. Its main function is to regulate the radial distribution of particle descending velocity and suppress the development of central funnel flow. Figure 2 shows planar screenshots of the three-dimensional shaft furnace models under different internal structural configurations. The flow distributor is placed at different axial positions in Cases 1–3, while the loosener is removed in Case 4. To improve the clarity and repeatability of the simulation setup, several key geometric parameters and the relative positions of the flow distributor and loosener are labeled in Figure 2. The corresponding case settings are listed in Table 2.

In this study, Case 1 was selected as the reference configuration according to the structural characteristics of a MIDREX-type shaft furnace, in which the flow distributor is located in the transition zone and the loosener is retained. The other cases were designed by modifying this reference configuration to represent typical structural configurations rather than to perform a full parametric optimization. Cases 2 and 3 were used to examine the effect of flow distributor position by moving it downward to the transition–cooling interface and the cooling zone, respectively. Case 4 was designed based on Case 1 by removing the loosener while keeping the same flow distributor position, so as to isolate the influence of the loosener. This case arrangement enables a focused comparison of the relative roles of flow distributor position and loosener configuration, although it does not cover all possible combinations of distributor positions and loosener settings.

## 3. Results and Discussion

### 3.1. Analysis of Particle Flow Velocity and Uniformity

**Figure 3:**
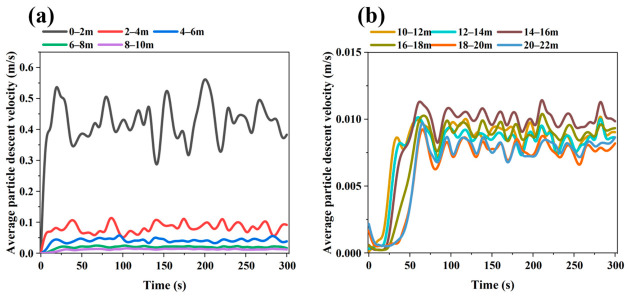
Temporal variation in particle descending velocity in regions at different heights of the shaft furnace when the flow distributor is located in the transition zone (Case 1): (**a**) 0–10 m; (**b**) 10–22 m.

**Figure 4:**
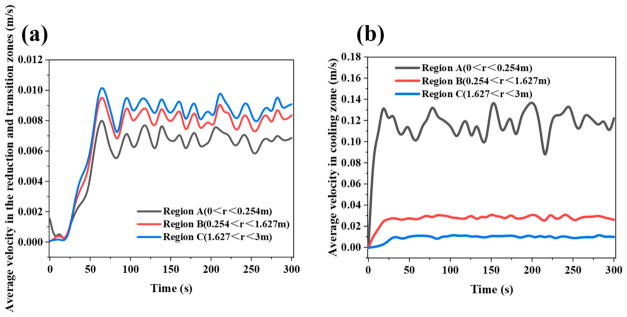
Temporal variation in particle descending velocity in different radial regions of the shaft furnace when the flow distributor is located in the transition zone: (**a**) reduction zone and transition zone; (**b**) cooling zone.

Figure 3 and Figure 4 show that, during the initial discharge stage after the start of the simulation, the average descending velocity of particles in each region changes markedly with time. This period corresponds to the transient adjustment of the burden bed from the initially packed state to a quasi-steady moving-bed state after continuous bottom discharge begins. As discharge proceeds, the velocities at all monitoring points gradually converge and fluctuate slightly around stable values, indicating that particle flow in the furnace evolves from a transient state to a quasi-steady state. Therefore, the subsequent analyses of particle velocity distribution, flow trajectories, and force behavior are all based on the stable operating stage, and the obtained results can more realistically reflect the regulating effects of different internal configurations on particle flow behavior in the shaft furnace. The radial monitoring regions used for velocity statistics are labeled in Figure 4. Region A represents the central region with 0 < r < 0.254 m, Region B represents the intermediate region with 0.254 < r < 1.627 m, and Region C represents the outer radial region with 1.627 < r < 3 m.

The velocity differences among the monitored regions are mainly caused by furnace geometry and the regulating effect of the flow distributor. As shown in Figure 3, the particle descending velocity is highest near the outlet of the conical section and decreases with increasing height, because the converging lower section accelerates particles toward the discharge outlet. In the vertical section, the velocity difference among different heights is relatively small, indicating dense moving-bed behavior after the quasi-steady state is reached. As shown in Figure 4, the central region in the vertical section has a lower descending velocity than the outer region due to the obstruction and redistribution effect of the flow distributor. In contrast, Region A in the conical section shows a higher velocity than Regions B and C because it includes particles close to the bottom outlet, where discharge-induced acceleration is strongest. Similar radial velocity differences and central preferential flow have also been reported in previous DEM studies of shaft furnaces [23,24].

**Figure 5:**
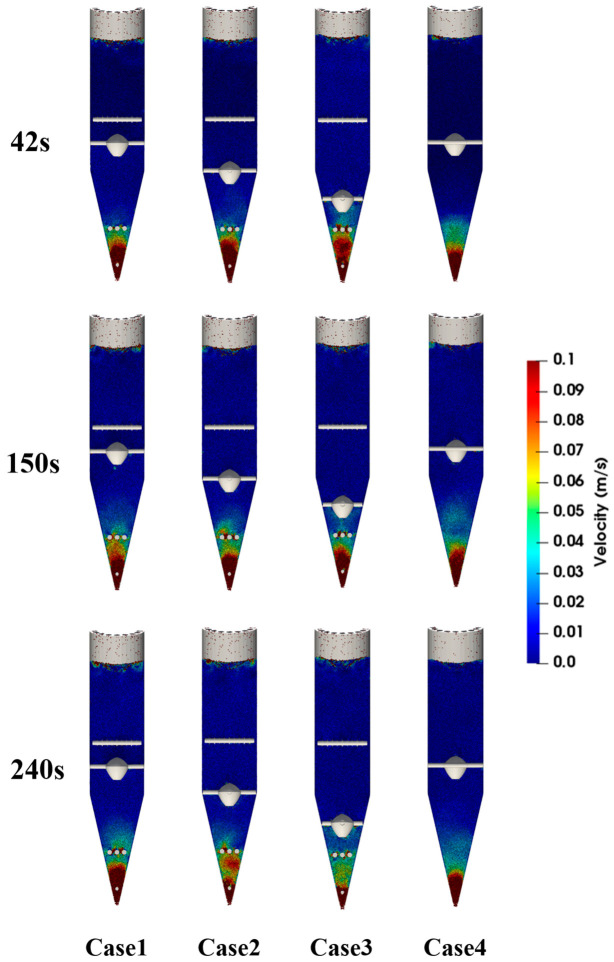
Contour maps of particle descending velocity in the shaft furnace at different time instants.

**Figure 6:**
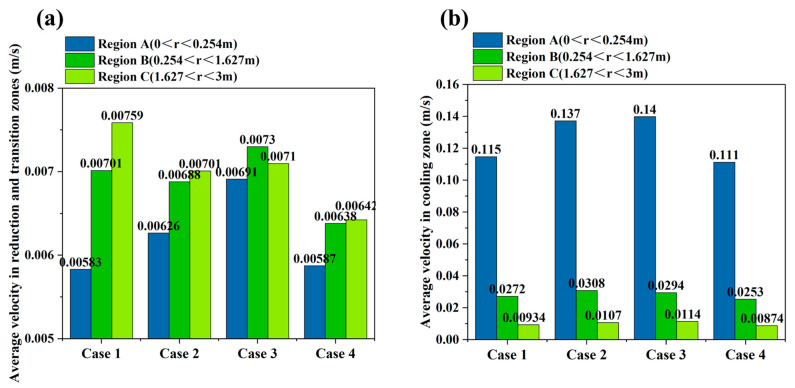
Comparison of the average descending velocity of burden particles under different cases: (**a**) reduction zone and transition zone; (**b**) cooling zone.

As shown in Figure 5 and Figure 6, the placement of the flow distributor has a significant effect on the distribution of particle descending velocity. Figure 5 shows that the high-velocity region is mainly concentrated near the central discharge channel and the lower conical section, whereas particles near the wall descend more slowly. This velocity distribution indicates that the converging geometry of the lower shaft furnace promotes preferential particle descent in the central region. Figure 6 further quantitatively compares the average descending velocity under different configurations. When the flow distributor is arranged in the cooling zone, the particles at the center of Region A exhibit the highest descending velocity, because the flow distributor has a weaker upstream obstruction effect on central particles. In contrast, when the flow distributor is arranged in the transition zone, central particles are diverted earlier, and their descending velocity is reduced. This indicates that moving the flow distributor upward to the transition zone can intervene earlier in the rapid descent of central particles, thereby weakening the development of central funnel flow. A similar phenomenon was reported by Tian et al. [24], who found that particles in the central region of a hydrogen-enriched shaft furnace descended faster than those near the wall, resulting in a V-shaped burden-flow pattern, while installing a diversion cone improved the uniformity of burden descent. In contrast, the loosener has a relatively small effect on the descending velocity of particles in the central region, indicating that the position of the flow distributor is the dominant factor in velocity regulation.

**Figure 7:**
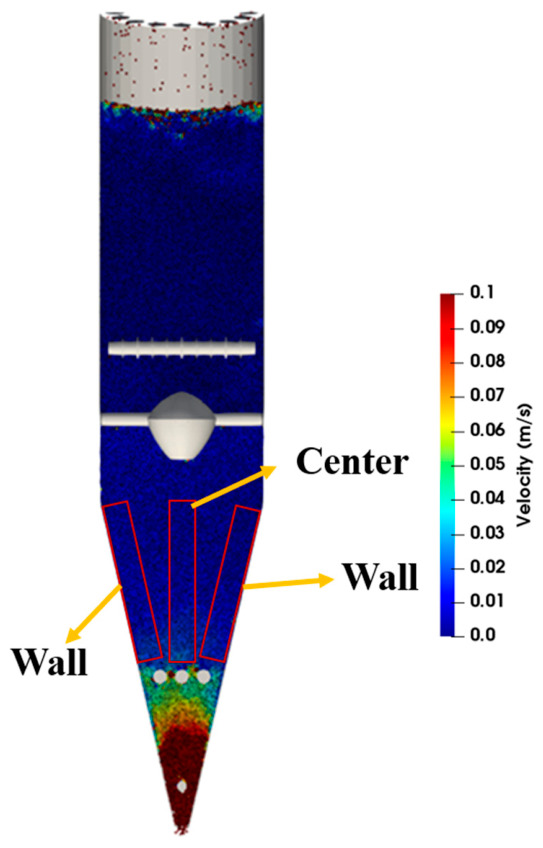
Schematic diagram of the division between the central region and the wall region in the conical section.

**Figure 8:**
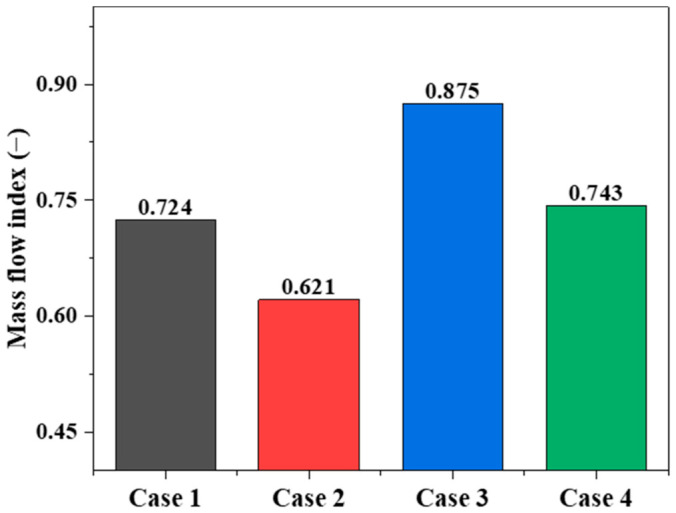
Comparison of the flow uniformity index under different cases.

Figure 7 presents the schematic division of the central region and the wall region in the conical section. A flow uniformity index is defined to quantitatively evaluate the uniformity of particle flow, and the corresponding formula is given as follows. Further analysis of the flow uniformity index shown in Figure 8 indicates that the position of the flow distributor has a more pronounced effect on particle flow uniformity in the conical section. When the flow distributor is located in the cooling zone, the flow uniformity index reaches 0.875, the highest among all cases. Compared with the case where the device is placed at the interface between the transition zone and the cooling zone, the index increases from 0.621 to 0.875, an improvement of 40.9%; compared with the case where it is placed in the transition zone, the index increases from 0.724 to 0.875, an improvement of 20.9%. This indicates that moving the flow distributor downward into the cooling zone can significantly reduce the flow difference between particles in the central and wall regions. On the other hand, when the flow distributor is moved upward from the interface between the transition zone and the cooling zone to the transition zone, the flow uniformity index increases from 0.621 to 0.724, an improvement of 16.6%, indicating that arranging the device in the transition zone can also effectively improve burden-flow uniformity. After removing the loosener, the flow uniformity index increases only from 0.724 to 0.743, an improvement of 2.6%, indicating that the effect of the loosener on flow uniformity is weaker than that of the flow distributor position. Therefore, from the perspective of particle-flow uniformity, placing the flow distributor in the cooling zone gives the most favorable result among the investigated cases. However, the final selection of the structural configuration should also consider particle stress distribution and degradation behavior, which are discussed in Section 3.3.

### 3.2. Analysis of Particle Trajectory Characteristics

**Figure 9:**
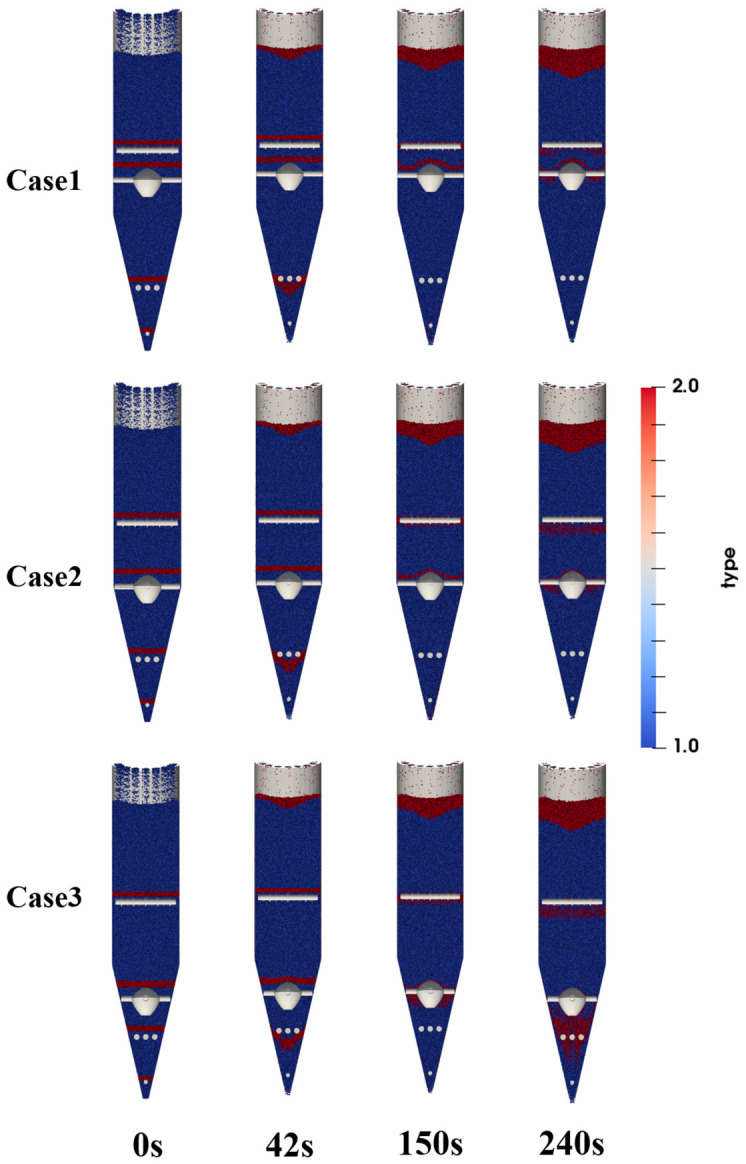
Snapshots of particle flow within the shaft furnace at different times.

To observe the influence of different flow distributor placements on particle trajectories, marked particle groups were used as tracer particles. Before discharge began (0 s), particles in the region above the flow distributor and at selected heights above the upper, middle, and lower looseners were marked in red. Their positions were recorded during the discharge process, and snapshots at different times were used to characterize the trajectory evolution of the marked particles, as shown in Figure 9. The results show that changes in the position of the flow distributor mainly affect the particle-flow behavior in the region above it. As the position of the flow distributor changes, the interface shape and downward trajectories of the marked particles above the flow distributor change significantly, indicating that the flow distributor has a pronounced diverting and perturbing effect on the upper particles. In contrast, the overall differences in the flow trajectories of the marked particles below the flow distributor, namely those above the middle and lower looseners, are not obvious and are only slightly affected by changes in the position of the flow distributor.

The reason is that the flow distributor mainly regulates particle flow by changing the local geometric boundary, and its effect is concentrated in the vicinity of the flow distributor and the region above it. After the position of the flow distributor is changed, the timing of contact between the upper particles and the flow distributor, the bypass paths, and the local diversion patterns all change, so the trajectories of the marked particles in this region differ markedly. In contrast, the motion of particles below the flow distributor is governed more by the overall discharge boundary conditions and the converging structure of the lower conical section. After being diverted by the flow distributor, the particles reconverge and continue to move downward. Therefore, different positions of the flow distributor have a relatively limited effect on particle trajectories in the region below it. This indicates that changing the position of the flow distributor mainly alters the local diversion behavior of upper particles rather than significantly changing the overall descending pattern of lower particles. This localized influence of internal structures is also consistent with the findings of Tian et al. [24], where changes in the diversion cone structure mainly altered the particle path and residence behavior near the cone region.

### 3.3. Analysis of Particle Stress and Powder Formation

In this study, the single-collision degradation model proposed in the literature was adopted to predict particle degradation behavior [17]. The proportion of particle mass loss caused by a single effective collision can be expressed by Equation (3). In the equation, *f_i_* is the degradation ratio corresponding to the *i*-th collision event, *E_i_* is the effective collision energy of the *i*-th particle collision, and k and *n* are empirical coefficients related to material properties. The model parameters *k* and *n* were taken from the experimental and numerical studies reported in the literature for pellets in a direct reduction shaft furnace, with values of 0.00029 and 1.16, respectively, and were implemented and applied in this work on that basis. The parameters *k* and *n* mainly affect the absolute value of the predicted degradation ratio, where *k* controls the proportional magnitude and *n* determines the nonlinear sensitivity to collision energy. Since the same *k* and *n* values were used for all cases, the relative comparison of powdering tendency among different structural configurations remains meaningful, although the calculated degradation ratios should be regarded as comparative indicators rather than exact industrial powder-generation values. During the steady operating stage, all effective collision events experienced by the particles were counted, and the single-collision degradation ratios were accumulated to obtain the cumulative degradation ratio of the particles during their movement through the shaft furnace.

(3) $$f_{i}=k{E_{i}}^{n}$$

**Figure 10:**
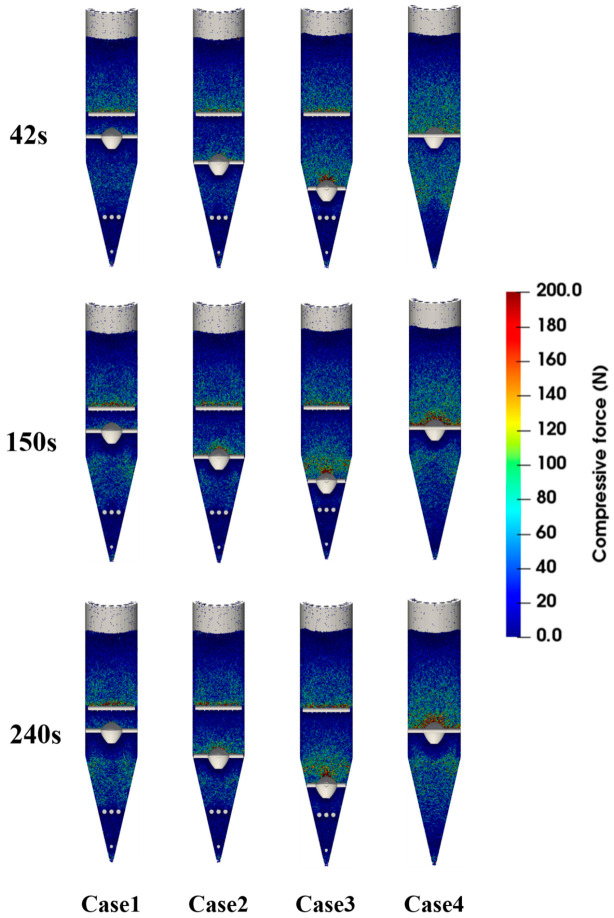
Contour maps of particle compressive-force distribution in the shaft furnace at different time instants.

**Figure 11:**
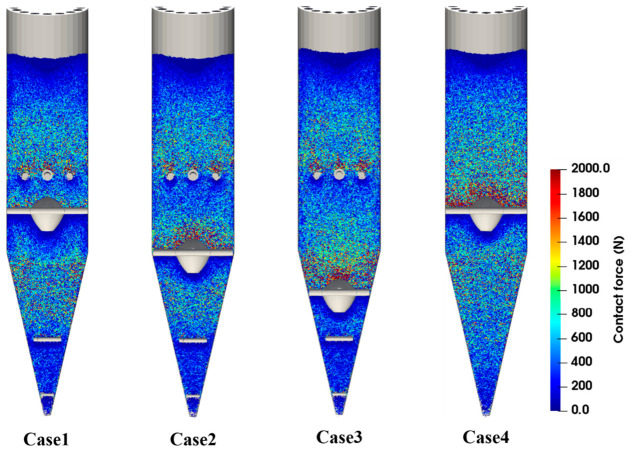
Force chain distribution within the shaft furnace at t = 300 s.

**Figure 12:**
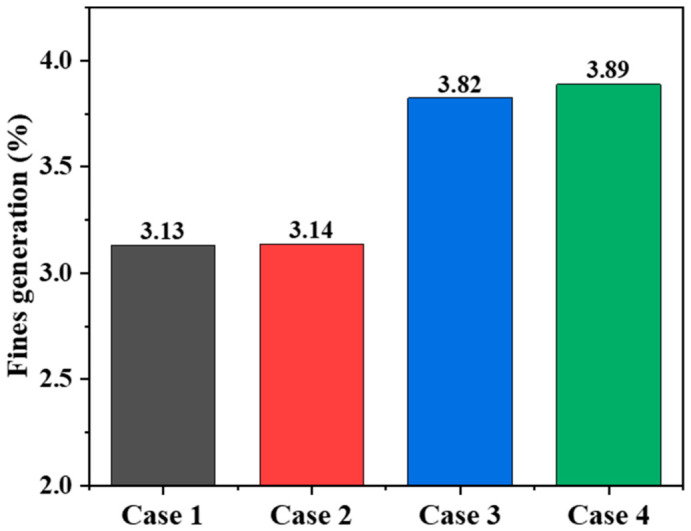
Statistics of average energy dissipation.

Figure 10, Figure 11 and Figure 12 show that the force distribution, force-chain structure, and energy dissipation characteristics of particles differ markedly under different operating conditions. Figure 10 presents the spatial distribution of particle compressive force, while Figure 11 visualizes the force-chain network and load-transfer paths within the burden bed. Thus, these two figures provide complementary information on force concentration and force transmission. When a high-velocity discharge channel forms in the central region of the furnace, continuous and concentrated high-stress chains are more likely to develop, especially near the central flow channel and the lower conical section. This results in higher local force peaks and intensified collision and extrusion effects, thereby increasing the risk of particle degradation. After optimizing the position of the flow distributor, the force level of particles in the central region decreases significantly, and the force-chain distribution gradually changes from local concentration to overall dispersion. This trend is consistent with our previous DEM study on hydrogen-based shaft furnace internal structures, which showed that structural optimization can affect compressive force distribution and collision energy dissipation, thereby influencing the mechanical stability of burden particles [23]. These results indicate that a reasonable internal configuration can improve load transfer within the burden bed and help reduce the tendency of particle breakage.

Combined with the statistical results of the degradation ratio, it can be seen that when the flow distributor is arranged in the cooling zone, although the flow uniformity index is the highest and reaches 0.875, the degradation ratio increases to 3.82%. This indicates that excessively strong local disturbance and structural constraint may cause contact stress to reconcentrate in the lower region, thereby increasing the risk of particle breakage. By contrast, when the flow distributor is located in the transition zone, the installation of the loosener reduces the degradation ratio from 3.89% to 2.97%, corresponding to a relative reduction of 23.7%. This result shows that although the loosener has a limited effect on improving particle-flow uniformity, it has clear advantages in relieving local extrusion, dispersing contact stress, and suppressing fines generation.

Therefore, the optimization of the internal structure of a hydrogen-based shaft furnace should not aim solely at maximizing particle-flow uniformity, but should also consider particle-force dispersion, smooth discharge, degradation control, and operational stability. Although placing the flow distributor in the cooling zone gives the highest particle-flow uniformity, it is also accompanied by a relatively high degradation ratio, indicating a greater tendency toward fines generation. In industrial operation, excessive fines may reduce bed permeability and increase the risk of unstable burden descent. In contrast, the transition-zone placement with the loosener retained is more beneficial for relieving local extrusion, dispersing contact stress, and suppressing fines generation. Therefore, among the four representative configurations investigated in this study, placing the flow distributor in the transition zone while retaining the loosener provides a more balanced compromise between particle-flow regulation and powder suppression.

It should be noted that the present study considers four representative structural configurations rather than a complete optimization over the full design space. The results therefore provide a comparative evaluation of typical flow distributor positions and loosener effects on particle-flow behavior. Although the limited number of cases does not allow a continuous trend analysis for all structural parameters, two clear tendencies can be identified: the flow distributor position mainly controls particle-flow uniformity and central particle descent, whereas the loosener contributes more to reducing particle degradation. In addition, no single configuration is superior in all respects. The cooling-zone placement gives the highest flow uniformity index of 0.875, but it also results in a relatively high degradation ratio and possible operational instability. By contrast, the transition-zone placement with the loosener retained provides a more balanced compromise between particle-flow regulation and powder suppression. Further work will consider more structural configurations and coupled gas–solid operating conditions for systematic optimization.

## 4. Conclusions

The conclusions were drawn from a combined evaluation of particle descending velocity, particle-flow uniformity, particle trajectory, force-chain distribution, energy dissipation, and particle degradation behavior. Among the investigated structural factors, the flow distributor position is the most important parameter controlling particle-flow regulation, while the loosener mainly contributes to degradation suppression.

(1) Particle flow in the shaft furnace exhibits obvious transient characteristics at the beginning of the calculation and then gradually enters a quasi-steady stage. The position of the flow distributor is the dominant factor affecting the distribution of particle descending velocity and particle-flow uniformity. When the flow distributor is arranged in the cooling zone, the uniformity index reaches the highest value of 0.875, representing increases of 40.9% and 20.9% compared with the transition–cooling interface and transition-zone configurations, respectively. When the flow distributor is moved upward from the transition–cooling interface to the transition zone, the uniformity index increases from 0.621 to 0.724, by 16.6%, indicating that placing the flow distributor in the transition zone can also effectively improve burden-flow uniformity.
(2) Changes in the position of the flow distributor mainly affect the flow trajectories of particles in the region above it, while exerting only a minor influence on the motion of particles below the flow distributor. As the position of the flow distributor changes, the interface shape and downward trajectories of the marked particles above the flow distributor change markedly, whereas the overall differences in the flow trajectories of the marked particles above the middle and lower looseners are not obvious. This indicates that the flow distributor mainly regulates the diversion and bypass behavior of upper particles by changing the local geometric boundary, and that its zone of influence is strongly localized.
(3) The risk of particle degradation is closely related to the degree of local force concentration. When the flow distributor is arranged in the cooling zone, although the burden flow is the most uniform, the degradation ratio rises to 3.82%, indicating that excessively strong local structural constraint causes contact stress to reconcentrate in the lower region. In contrast, when the flow distributor is arranged in the transition zone, installing the loosener reduces the degradation ratio from 3.89% to 2.97%, a relative decrease of 23.7%, indicating that the loosener plays a significant role in dispersing load transfer, relieving local extrusion, and suppressing fines generation.
(4) Considering multiple performance indicators, including particle-flow uniformity, particle degradation, force dispersion, and operational stability, no single configuration is superior in every respect. Placement of the flow distributor in the cooling zone raises the flow uniformity index to the highest value of 0.875, but it is accompanied by a relatively high degradation ratio of 3.82% and possible operational instability. Although placement in the transition zone yields slightly lower particle-flow uniformity, it provides a better compromise between burden-flow regulation and powder suppression. In particular, when the loosener is retained, good burden-flow organization can be maintained while the degradation ratio is kept at a relatively low level of 2.97%. Therefore, among the four representative configurations investigated in this study, placing the flow distributor in the transition zone while retaining the loosener is considered a more balanced structural configuration.

The present work should be regarded as a comparative DEM study for preliminary structural screening rather than a direct quantitative prediction of industrial furnace operation. The enlarged pellet diameter of 96 mm reduces computational cost but limits the quantitative scalability of the calculated velocity, force, and degradation results to real pellet-size conditions. However, because all cases use the same particle size, contact parameters, and boundary conditions, the relative differences among different structural configurations remain meaningful for identifying design tendencies. The industrial relevance of this study lies in revealing the trade-off between particle-flow uniformity and degradation suppression and providing preliminary guidance for internal-structure optimization. Future work will improve the industrial applicability of the model through smaller-particle DEM simulations, cold physical model validation, comparison with industrial operation data, and coupled CFD–DEM modeling including gas flow, heat transfer, and reduction reactions.

## Figures and Tables

**Table 1:** Material properties and simulation parameters [17,21,22,23].

Parameters		Pellet	Wall
Density, ρ (kg/m^3^)		3948	7850
Young’s modulus, E (MPa)		16	7000
Poisson’s ratio, υ (−)		0.25	0.3
Restitution coefficient, e (−)	Pellet	0.39	0.48
Static friction, μ_s_ (−)	Pellet	0.5	0.49
Rolling friction, μ_r_ (−)	Pellet	0.25	0.21

**Table 2:** Simulation case settings.

	Detailed Description
Case 1	Flow distributor placed in the transition zone
Case 2	Flow distributor placed at the interface between the transition zone and the cooling zone
Case 3	Flow distributor placed in the cooling zone
Case 4	Flow distributor placed in the transition zone, with the loosener removed

## Data Availability

The original contributions presented in this study are included in the article. Further inquiries can be directed to the corresponding authors.
